# Structural Change from Nonparallel to Parallel G-Quadruplex Structures in Live Cancer Cells Detected in the Lysosomes Using Fluorescence Lifetime Imaging Microscopy

**DOI:** 10.3390/ijms232415799

**Published:** 2022-12-13

**Authors:** Ting-Yuan Tseng, Chiung-Lin Wang, Ta-Chau Chang

**Affiliations:** Institute of Atomic and Molecular Sciences, Academia Sinica, Taipei 10617, Taiwan

**Keywords:** G-quadruplexes, structural conversion, parallel G4s in the lysosomes, time-gated fluorescence lifetime imaging microscopy, *o*-BMVC fluorescent probe

## Abstract

Time-gated fluorescence lifetime imaging microscopy with the *o*-BMVC fluorescent probe provides a visualizing method for the study of exogenous G-quadruplexes (G4s) in live cancer cells. Previously, imaging results showed that the parallel G4s are accumulated and that nonparallel G4s are not detected in the lysosomes of CL1-0 live cells. In this work, the detection of the G4 signals from exogenous GTERT-d(FN) G4s in the lysosomes may involve a structural change in live cells from intramolecular nonparallel G4s to intermolecular parallel G4s. Moreover, the detection of the G4 signals in the lysosomes after the 48 h incubation of HT23 G4s with CL1-0 live cells indicates the occurrence of structural conversion from the nonparallel G4s to the parallel G4s of HT23 in the live cells. In addition, the detection of much stronger G4 signals from ss-GTERT-d(FN) than ss-HT23 in the lysosomes of CL1-0 live cells may be explained by the quick formation of the intermolecular parallel G4s of ss-GTERT-d(FN) and the degradation of ss-HT23 before its intramolecular parallel G4 formation. This work provides a new approach to studying G4-lysosome interactions in live cells.

## 1. Introduction

During the last two decades, guanine-quadruplexes (G4s) have received increasing attention because of the association of G4s with genome instability, genetic diseases, and cancer progression [1,2]. The existence of G4s in cells could be supported by the existence of proteins that are able to bind and unfold G4 structures [3]. Two G4-specific monoclonal antibodies, BG4 [4] and 1H6 [5], provided convincing evidence using immunofluorescence microscopy to support the presence of G4s in the metaphase chromosomes and fixed cells. We further introduced fluorescence lifetime imaging microscopy (FLIM) of the 3,6-bis(1-methyl-2-vinylpyridinium) carbazole diiodide (*o*-BMVC) fluorescent probe to detect G4s in both live and fixed cells [6,7]. Notably, *o*-BMVC showed a higher binding affinity to telomeric G4s than to duplex DNA by nearly two orders of magnitude [6]. Moreover, the fluorescent decay times of *o*-BMVC were measured to be ≥2.4 ns upon binding to 20 different G4 structures and <1.6 ns upon interaction with 10 different non-G4 structures, including single-stranded (ss) DNA and duplex DNA [7]. Thus, the 2.4 ns could be used as a time threshold to separate the FLIM image into two temporal components for visualizing the G4s. Particularly, the existence of telomeric G4s was specifically identified in the metaphase chromosomes and in the fixed cancer cells using antisense DNA [8]. Given that more G4 foci were detected in the cancer cells than in normal cells [7,9,10], time-gated FLIM image of *o*-BMVC foci was further applied to the cancer diagnosis of fine-needle aspiration of thyroid nodules [11]. Such studies strongly supported not only the existence but also the importance of G4s in cancer cells.

The details of the G4 structures and dynamics have been extensively studied in vitro [12,13,14,15,16], but much less so in live cells. This is because the signals from very small amounts of G4s are easily blocked by the overwhelming signals from duplex DNA and other sources in live cells. Given that several G-rich oligonucleotides (GROs), such as AS1411 [17], T40214 [18], HT24 [19], and PU27 [20], were studied as anticancer agents to inhibit cancer cell growth, we previously used imaging methods to examine the cellular uptake of these exogenous G4s in CL1-0 live cells after a 2 h incubation [21,22]. The imaging results showed that the parallel G4 structures of AS1411, T40214, and PU22 are easily detected in the lysosomes, but the nonparallel G4 structures of HT23 and TBA are hardly detected in the lysosomes of CL1-0 live cells. In addition, the folding time of the ss-CMA to the G4 structure and the unfolding time of the CMA G4s after the addition of the anti-CMA in live cells were to some extent similar to that measured in K^+^ solution [22].

Very recently, we found that a GRO, d(G_3_AG_4_CTG_3_AG_3_), named GTERT-d(FN) could initially form intramolecular nonparallel G4 structures but eventually convert to intermolecular parallel G4 structures in a 100 mM K^+^ solution [23]. It is crucial to examine whether such structural change of exogenous GTERT-d(FN) can be detected in the lysosomes of CL1-0 live cells. In addition, Miyoshi et al. [24] have used polyethylene glycol (PEG) to mimic cellular crowding environment and found that PEG can induce structural change of telomeric G4s. Heddi and Phan [25] further reported that four different nonparallel G4 structures of telomeric G-rich sequences all converted to the parallel G4 structure under the PEG condition. It is important to examine whether the structural conversion of exogenous HT23 from nonparallel G4s to parallel G4s can be detected in the lysosomes of CL1-0 live cells.

## 2. Results

### 2.1. Temperature Effect on the Structural Conversion of GTERT-d(FN) G4s in Solution

**Figure 1:**
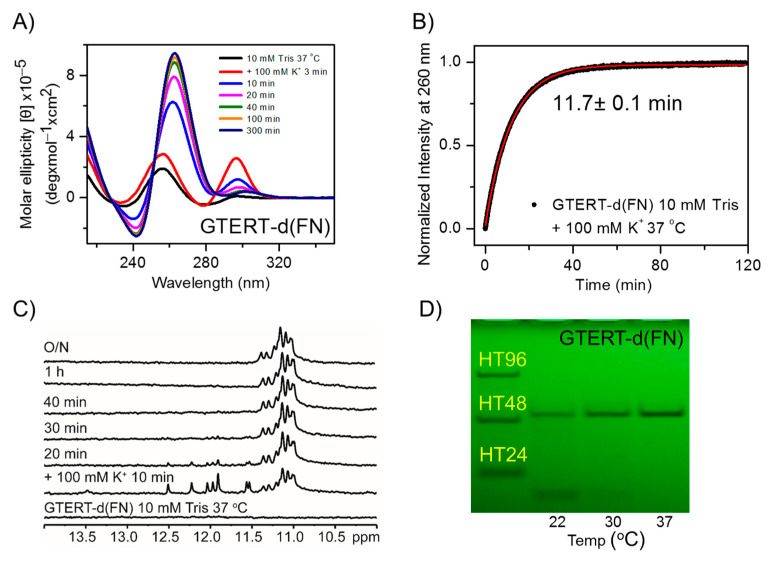
Structural conversion of GTERT-d(FN) in a K^+^ solution. (**A**) Time-dependent CD spectra of GTERT-d(FN) in a 100 mM K^+^ solution at 37 °C. (**B**) The arising time of G4-2 signals is 11.7 ± 0.1 min, obtained from a single exponential fitting at 265 nm. (**C**) Time-resolved imino proton spectra of GTERT-d(FN) recorded at 0, 10, 20, 30, 40, and 60 min together with overnight after the addition of 100 mM K^+^. (**D**) UV shadowing of gel assay of GTERT-d(FN) in a 10 mM Tris buffer after the 1 h addition of a 100 mM K^+^ at 22 °C (lane 1), 30 °C (lane 2), and 37 °C (lane 3) at a DNA concentration of 80 μM.

Previously, we reported the finding of a spectral change of GTERT-d(FN) in 100 mM K^+^ solution at room temperature (~22 °C) [23]. Given that cellular experiments are generally conducted at 37 °C, we studied spectral change of GTERT-d(FN) in a 100 mM K^+^ solution at 37 °C by time-dependent CD spectra (Figure 1A). Initially, the detection of a major CD band at 295 nm suggests that the formation of the non-parallel G4s is the main component. Subsequently, the decrease of the 295 nm band associated with the increase of the 265 nm band suggests structural change from the nonparallel G4s (named G4-1) to parallel G4s (named G4-2). The conversion time is obtained by measuring the increase of the CD signal at 265 nm, which is fitted to a single exponential function with a time constant of 11.7 ± 0.1 min (Figure 1B). Notably, the conversion time is measured to be 57.4 ± 1.4 min at 30 °C and 318 ± 3.4 min at 22 °C (Figure S1). In addition, NMR spectra of GTERT-d(FN) show two distinct components: the G4-1 component, characterized by several fine imino proton signals between 11.5–12.5 ppm appears initially but disappears within 1 h, and the G4-2 component, characterized by several fine imino proton signals on top of a broad band in the region of 11.0–11.5 ppm, increases due to the conversion of the G4-1 component in 100 mM K^+^ solution at 37 °C (Figure 1C). Notably, the NMR results clearly show that the initial component is the G4-1 formation at room temperature (Figure S1). Furthermore, PAGE assays show an intramolecular band and an intermolecular band at 22 °C, but only an intermolecular band at 37 °C for GTERT-d(FN) after the 1 h addition of 100 mM K^+^ (Figure 1D). This finding is consistent with the 11.7 min at 37 °C and 318 min at 22 °C for structural change from the intramolecular nonparallel G4s (G4-1) to the intermolecular parallel G4s (G4-2) of GTERT-d(FN) in 100 mM K^+^ solution.

### 2.2. Time-Gated FLIM Images of the Exogenous GTERT-d(FN) G4s in CL1-0 Live Cells

**Figure 2:**
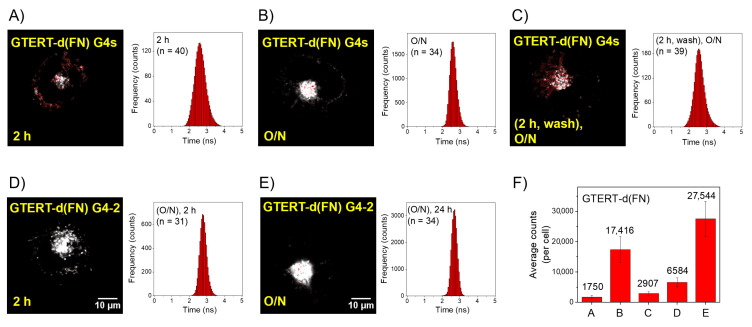
Time-gated FLIM images of exogenous GTERT-d(FN) G4s in CL1-0 live cells. Time-gated FLIM images of 5 μM *o*-BMVC mixed with 15 μM GTERT-d(FN) G4s incubated with CL1-0 live cells for 2 h (**A**) and overnight (O/N) (**B**). The incubation of 5 μM *o*-BMVC mixed with 15 μM GTERT-d(FN) G4s with CL1-0 live cells for 2 h followed by washing twice with PBS and then cultivated in a new medium for O/N culture (**C**). The incubation of 5 μM *o*-BMVC mixed with 15 μM GTERT-d(FN) G4-2 with CL1-0 live cells for 2 h (**D**) and overnight (O/N) (**E**). Here a decay time ≥ 2.4 ns is characterized in white and a decay time < 2.4 ns is characterized in red. The histograms of the average photon counts per cell with a decay time ≥ 2.4 ns for each case (**F**).

To examine whether the intermolecular parallel G4s can be detected in the lysosomes of CL1-0 live cells, Figure 2A,B shows the time-gated FLIM images together with the histograms of average photon counts of *o*-BMVC fluorescence per cell after the incubation of 5 μM *o*-BMVC mixed with 15 μM GTERT-d(FN) G4s with CL1-0 live cells for 2 h and overnight, respectively. Here a decay time ≥ 2.4 ns is presented in white and a decay time < 2.4 ns is presented in red. The images show significantly more G4 signals in the lysosomes after the overnight incubation than after the 2 h incubation, indicating the accumulation of the intermolecular parallel G4s in the lysosomes of live cells. Notably, there are two pathways for the detection of parallel G4-2 signals in the lysosomes, which could be due to either the parallel G4-2 converted from the nonparallel G4-1 in the medium followed by the cellular uptake (pathway 1) or the cellular uptake of nonparallel G4-1 followed by the structural conversion to the parallel G4-2 in CL1-0 live cells (pathway 2).

To identify the structural conversion of GTERT-d(FN) G4 in live cells, the samples were washed with PBS twice after the 2 h incubation in 5 μM *o*-BMVC and 15 μM GTERT-d(FN) G4 with CL1-0 live cells and then cultivated in a new medium overnight. Figure 2C shows the time-gated FLIM image together with the histogram of the average photon count of *o*-BMVC fluorescence per cell obtained from such samples. The images show more G4 signals in the lysosomes of Figure 2C than Figure 2A, suggesting that the structural conversion from the nonparallel G4-1 to parallel G4-2 of exogenous GTERT-d(FN) is possible in the live cells.

Since the parallel G4-2 formation of GTERT-d(FN) is eventually predominated in a K^+^ solution overnight, the incubation of the G4-2 samples with CL1-0 live cells allows us to verify the detection of intermolecular parallel G4s in the lysosomes of live cells. Figure 2D,E shows the time-gated FLIM images together with the histograms of average photon counts of *o*-BMVC fluorescence per cell after the incubation of 5 μM *o*-BMVC mixed with 15 μM GTERT-d(FN) G4-2 with CL1-0 live cells for 2 h and overnight, respectively. Figure 2F shows the plots of the average photon counts per cell with the decay time ≥ 2.4 ns for each case, which are measured from the histograms in Figure 2A–E. The difference of average photon counts between case D and case A is larger than that between case C and case A, indicating that pathway one primarily contributes to the detection of parallel G4s in the lysosomes of CL1-0 live cells. Considering the lower contribution to the G4 signals in the lysosomes from pathway 2 and the large deviation in the photon counts of *o*-BMVC fluorescence measured in live cells, it is difficult to measure a reliable rate for the structural conversion from the nonparallel G4s to the parallel G4s of GTERT-d(FN) in live cells.

### 2.3. Imaging Study of the Exogenous HT23 G4s in CL1-0 Live Cells

**Figure 3:**
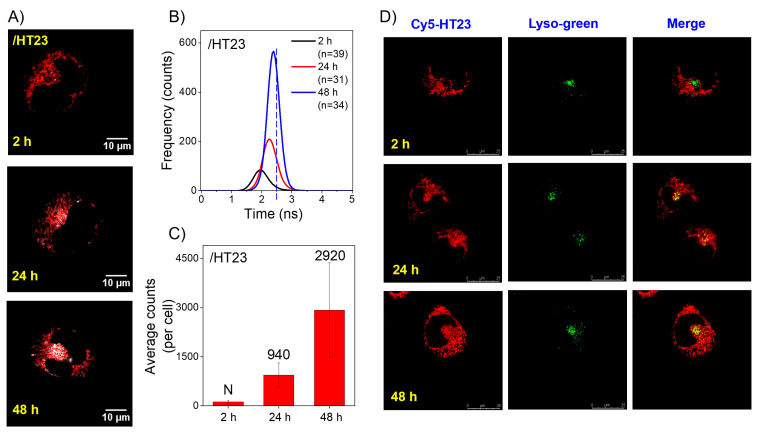
Imaging study of exogenous HT23 G4s in CL1-0 live cells. Time-gated FLIM images of 5 μM *o*-BMVC mixed with 15 μM HT23 G4s incubated with CL1-0 live cells for 2 h, 24 h, and 48 h (**A**). The corresponding plots of average photon counts of *o*-BMVC fluorescence per cell after 2 h, 24 h, and 48 h incubation of HT23 G4s with CL1-0 live cells (**B**). Histogram of the average photon counts per cell with a decay time ≥ 2.4 ns for each case, where N denotes negligible (**C**). Confocal images of Cy5-HT23 G4s incubated with CL1-0 live cells for 2 h, 24 h, and 48 h (**left**), and then stained by LysoTracker green at a concentration of 50 nM and incubated with the cells for 15 min (**middle**) together with their merges (**right**) (**D**).

The detection of the intermolecular parallel G4s in the lysosomes due to structural conversion from the intramolecular nonparallel G4s of exogenous GTERT-d(FN) in live cells leads us to examine whether the conversion from the nonparallel G4s to parallel G4s of human telomeric GROs can be detected in the lysosomes of CL1-0 live cells. Figure 3A shows the time-gated FLIM images after the incubation of 5 μM *o*-BMVC mixed with 15 μM HT23 G4s with CL1-0 live cells for 2 h, 24 h, and 48 h. The images show negligible G4 signals, slight G4 signals, and marked G4 signals in the lysosomes after the 2 h, 24 h, and 48 h incubations, respectively. The corresponding histograms of their average photon counts of *o*-BMVC fluorescence per cell are shown in Figure 3B, and the plots of their average photon counts per cell with the decay time ≥ 2.4 ns are shown in Figure 3C. Here, the detection of the parallel G4s in the lysosomes indicates that the nonparallel G4s of exogenous HT23 can convert to parallel G4s in CL1-0 live cells.

In addition, confocal microscopy has been applied to visualize the cellular uptake of fluorophore-labeled GROs. Previously, the CD spectra of Cy5-HT23 showed no appreciable change on the nonparallel G4 structure of HT23 by covalently linked Cy5 dye in a 150 mM K^+^ solution [22]. Figure 3D (left) shows the confocal images of Cy5-HT23 G4s incubated with CL1-0 live cells for 2 h, 24 h, and 48 h. The Cy5 fluorescence is hardly detected in the nucleus but is observed in the cytoplasm of CL1-0 live cells. The LysoTracker green was added to determine whether the nonparallel G4s of Cy5-HT23 could be detected in the lysosomes of CL1-0 cells (Figure 3D, central). The confocal image shows no appreciable merge between LysoTracker green and Cy5 red after the 2 h incubation (Figure 3D, right), confirming the absence of the nonparallel G4s of Cy5-HT23 in the lysosomes of CL1-0 live cells. Importantly, the confocal images clearly show some merges of LysoTracker green with Cy5 red after the 24 h incubation, and even more merges after the 48 h incubation.

Here, the late detection of the parallel G4s of HT23 in the lysosomes from the incubation of nonparallel HT23 G4s in both time-gated FLIM and confocal images indicates that the structural conversion from nonparallel G4s to parallel G4s of HT23 indeed occurs in CL1-0 live cells. In addition, the distribution of Cy5-HT23 in the cytoplasm, but not in the lysosomes, after the 2 h incubation suggests that the lysosomes are unlikely the organelle for the structural conversion from nonparallel G4s to parallel G4s of Cy5-HT23. Thus, the late detection of HT23 G4s in the lysosomes is likely due to the conversion from nonparallel G4s to parallel G4s during endocytosis and then fusion with lysosomes.

### 2.4. Time-Gated FLIM Images of the Exogenous ss-GROs in CL1-0 Live Cells

**Figure 4:**
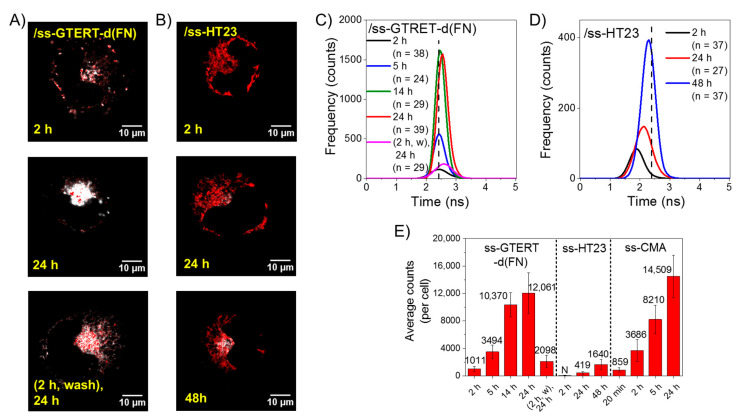
Time-gated FLIM images of exogenous ss-GROs in CL1-0 live cells. Time-gated FLIM images of 5 μM *o*-BMVC mixed with 15 μM ss-GTERT-d(FN) incubated with CL1-0 live cells for 2 h and overnight ((**A**), **top** and **middle**). In addition, the samples were washed with PBS twice after the 2 h incubation of 5 μM *o*-BMVC and 15 μM ss-GTERT-d(FN) with CL1-0 live cells and then cultivated in a new medium overnight ((**A**), **bottom**). The incubation of 5 μM *o*-BMVC mixed with 15 μM ss-HT23 with CL1-0 live cells for 2 h, 24 h, and 48 h (**B**). The corresponding plots of their average photon counts of *o*-BMVC fluorescence per cell (**C**,**D**), respectively. The histograms of the average photon counts per cell with a decay time ≥ 2.4 ns after the incubation of ss-GTERT-d(FN), ss-HT23, and ss-CMA with CL1-0 live cells at different times, where N denotes negligible (**E**).

We now study the time-gated FLIM images after the incubation of 5 μM *o*-BMVC mixed with 15 μM ss-GTERT-d(FN) with CL1-0 live cells as a function of time. Figure 4A (top and middle) shows the images detected after the incubation times of 2 h and 24 h. Given that the ss-GROs cannot form G4s without the salts in the medium, the detection of the G4 signals is due to the cellular uptake of the ss-GROs followed by the parallel G4 formation in the live cells. In addition, the samples were washed with PBS twice after the 2 h incubation of 5 μM *o*-BMVC and 15 μM ss-GTERT-d(FN) with CL1-0 live cells and then cultivated in a new medium overnight (Figure 4A, bottom). Figure 4A (bottom) shows more G4 signals in the lysosomes than Figure 4A (top), suggesting that the accumulation of the parallel G4s in the lysosomes can be described by the endocytosis of ss-GTERT-d(FN) and the parallel G4 formation in the cytoplasm.

We have further examined the time-gated FLIM images of 5 μM *o*-BMVC mixed with 15 μM ss-HT23 after the 2 h, 24 h, and 48 h incubations with CL1-0 live cells (Figure 4B). The detection of parallel G4s in the lysosomes after incubation with ss-HT23 is much less than the incubation of ss-GTERT-d(FN) with CL1-0 live cells. This is probably due to the degradation of ss-HT23 before its parallel G4 formation in the lysosomes. The corresponding histograms of their average photon counts of *o*-BMVC fluorescence per cell are shown in Figure 4C,D, together with the plots of their average photon counts per cell with the decay time ≥ 2.4 ns are shown in Figure 4E. For comparison, the plots of the average photon counts of *o*-BMVC fluorescence per cell after the incubation of ss-CMA with CL1-0 live cells for 20 min, 2 h, 5 h, and 24 h are also shown in Figure 4E, which were obtained from the previous results [22].

The detection of parallel G4s in the lysosomes after the incubation of ss-GTERT-d(FN) and ss-HT23 in live cells indicates that their parallel G4s can be formed in CL1-0 live cells. Notably, the average photon counts of ss-GTERT-d(FN) is significantly more than that of ss-HT23 and is similar to that of ss-CMA in the lysosomes of live cells after the 24 h incubation. The previous study showed that the arising time of the CD signal at 265 nm of CMA is about 80 s in a 100 mM K^+^ solution, and the detection of marked G4 signals in the lysosomes from the cellular uptake of ss-CMA is within 20 min [22]. Thus, the detection of the parallel G4s from ss-CMA is likely determined by the cellular uptake of ss-CMA in live cells. Notably, the normalized G4 signals detected in the lysosomes after the incubation of ss-CMA with CL1-0 live cells at 20 min, 2 h, 5 h, and 24 h can be fitted to a single exponential function with a single constant, suggesting that the increase of G4 signals detected in the lysosomes can be described by one major mechanism.

As for the incubation of ss-GTERT-(FN) with CL1-0 live cells, the G4 signals detected in the lysosomes after the 2 h incubation of ss-GTERT-d(FN) is much less than that of ss-CMA. However, the G4 signals detected in the lysosomes after the 24 h incubation of ss-GTERT-d(FN) is similar to that of ss-CMA. Notably, the arising time of the CD signal at 265 nm is about 12 min for GTERT-d(FN) in 100 mM K^+^ solution at 37 °C, which is much longer than the 80 s for CMA in 100 mM K^+^ solution at room temperature. Such differences in folding time may result in different G4 signals detected in the lysosomes after the 2 h incubation with CL1-0 live cells. The details of the similar amounts of G4 signals detected in the lysosomes after the 24 h incubation with CL1-0 live cells are not clear at present, and they warrant further investigation.

## 3. Discussion

The results show that the conversion time from intramolecular nonparallel G4s to intermolecular parallel G4s of GTERT-d(FN) is 318 min at 22 °C and 11.7 min at 37 °C in a 100 mM K^+^ solution, indicating that the conversion time for the structural change of GTERT-d(FN) is very sensitive to the experimental temperature. In addition, the Na^+^ concentration (20–140 mM) is higher than the K^+^ concentration (2–50 mM) in the lysosomes [26]. As a result, we have investigated the effect of Na^+^ on GTERT-d(FN) G4s in solution (Figure S2). The CD spectra of GTERT-d(FN) after adding 100 mM Na^+^ into 100 mM K^+^ or after adding 10 mM K^+^ into 100 mM Na^+^ in solution show that the CD spectra are mainly determined by the presence of K^+^. This finding is consistent with the previous study of HT23 after adding 150 mM K^+^ to 150 mM Na^+^ [16], indicating that the G4 structures are more stable in K^+^ solution than in Na^+^ solution. Furthermore, a time-dependent CD spectra in Na^+^ solution suggests that the Na^+^ has no appreciable effect on the parallel G4 structures detected in the lysosomes of CL1-0 live cells.

The detection of parallel G4s of GTERT-d(FN) in the lysosomes indicates that the intermolecular parallel G4s can accumulate in the lysosomes of CL1-0 live cells. Further study of the confocal images of a colocalization of Cy5-GTERT-d(FN) G4-2 with LysoTracker green supports the accumulation of the parallel G4s in the lysosomes of CL1-0 live cells (Figure S3). Notably, the LysoTracker green stains both lysosomes and late endosomes. This is because the endosome is a vesicle that contains internalized materials during endocytosis, and the lysosome is another vesicle that contains hydrolytic enzymes for the degradation of macromolecules. Endosomes and lysosomes interact through two distinct pathways: kiss-and-run and direct fusion [27]. Thus, one should refer to the lysotracker compartment as endo-lysosomal. Because late endosomes and lysosomes share many properties, in most cases we refer to both organelles as lysosomes, except when a distinction is necessary [28]. Given that cellular experiments are generally conducted at 37 °C, the detection of the G4 signals in the lysosomes after a 2 h incubation of GTERT-d(FN) G4s with CL1-0 live cells is primarily due to cellular uptake of parallel G4s caused by structural conversion in the medium. In addition, the detection of the marked G4 signals in the lysosomes following a 2 h incubation of ss-GTERT-d(FN) with CL1-0 live cells may be explained by the rapid formation of parallel G4s during endocytosis.

The important finding is the detection of the parallel G4s of HT23 in the lysosomes, indicating the occurrence of the structural conversion of HT23 from the nonparallel G4 structure to the parallel G4 structure in CL1-0 live cells. In comparison with the study of ss-GTERT-d(FN), the much less detectable parallel G4 signals from ss-HT23 is likely due to the degradation of ss-HT23 before its parallel G4 formation. Lysosomes have long been considered as the digestion machines for cellular clearance, degrading waste disposal both inside of cells via autophagy and outside of cells via endocytosis [29,30]. In addition, the parallel G4 signals detected in the lysosomes after the 24 h incubation of the HT23 G4s and GTERT-d(FN) G4s are greater than the signals detected after the 24 h incubation of the ss-HT23 and ss-GTERT-d(FN), respectively, suggesting that the degradation of the ss-GROs is easier than that of the GRO G4s in the lysosomes of live cells. Notably, the study of G4-lysosome interactions in live cells is just beginning. To our knowledge, this is the first report on the structural conversion of GROs from nonparallel G4s to parallel G4s in live cells. The current study provides a new approach to studying the dynamic nature of G4-lysosome interactions in live cells at a molecular level.

Recently evidence suggests that lysosomes play a central role in many cellular processes, including degradation, metabolic signaling, gene regulation, and quality control [29,30]. Defects in the degradation process result in lysosomal dysfunction and lysosomal storage disorders (LSD) that could cause several neurodegenerative and metabolic diseases, as well as cancer [30]. At present, it is not clear why the parallel G4s, but not the nonparallel G4, can be accumulated in the lysosomes of CL1-0 live cells. Notably, lysosomes have been much less characterized. For example, the lysosomal membrane comprises hundreds of integral and peripheral proteins, many of which have unknown functions [30]. Here we consider that the accumulation of the parallel G4s in the lysosomes may be used to uncover the fundamental mechanism of LSD. In addition, it is possible to use the parallel G4s as a drug carrier to treat lysosomal-related diseases for therapeutic purposes.

Lysosomes were considered as garbage cans and mitochondria were considered power house for a long time. They were independent and physically separated organelles. Recent studies of mitochondria-lysosome interactions demonstrated that they mutually regulate intracellular metabolism and maintain proper cell homeostasis. In addition, defective mitochondria-lysosome interaction may lead to neurodegenerative diseases [31]. Very recently, a novel fluorescent probe was used to study localization and dynamic tracking of mitochondria-lysosome interactions in the live cells [32]. Given that the nonparallel G4s of Cy5-HT23 mainly localize in the mitochondria of CL1-0 living cells [21], it is interesting to examine whether the detection of the parallel G4s in lysosomes and the nonparallel G4s in the mitochondria of Cy5-HT23 is associated with the mitochondria-lysosome interactions in the live cells.

## 4. Materials and Methods

### 4.1. DNA Preparation

DNA oligonucleotides were purchased from Bio Basic (Markham, ON, Canada) and dissolved in 10 mM Tris (pH 7.5). They were then subjected to heat denaturation at 95 °C for 10 min and annealed to room temperature at a rate of 1 °C/min. The annealed oligonucleotides were stored at 4 °C overnight until the further experiments. The DNA concentrations were determined using a UV-Vis absorption spectrometer (Implen, Munich, Germany).

### 4.2. Circular Dichroism (CD)

CD experiments were conducted using a spectropolarimeter (J-815, Jasco, Tokyo, Japan) with a bandwidth of 2 nm, a scan speed of 50 nm/min, and a step resolution of 0.2 nm over a spectral range of 210–350 nm. The DNA concentration in each sample was 100 μM dissolved in 10 mM Tris (pH 7.5), and a stock solution of 3 M KCl (Sigma-Aldrich, St. Louis, MO, USA) was added to the DNA samples to attain a final K^+^ concentration. The observed signals were baseline subtracted.

### 4.3. Nuclear Magnetic Resonance (NMR) Spectroscopy

NMR experiments were performed using an AVIII 500 MHz spectrometer (Bruker, Rheinstetten, Germany) equipped with a prodigy probehead at a specific temperature. One-dimensional imino proton NMR spectra were recorded using a WATERGATE for water suppression. The strand concentrations of the NMR samples were 100 μM, containing 10% D_2_O in 10 mM Tris (pH 7.5) or 100 mM K^+^ conditions, with an internal reference of 0.01 mM 4,4-dimethyl-4-silapentane-1-sulfonic acid (DSS).

### 4.4. Polyacrylamide Gel Electrophoresis (PAGE)

PAGE was conducted using 16% polyacrylamide and 0.5× TBE gels in the presence of a 20 mM K^+^ concentration. PAGE was conducted at 175 V for 225 min at 10 °C. The gels were then photographed under ultraviolet (UV) light at 254 nm using a digital camera.

### 4.5. Cell Cultures

CL1-0, a human lung carcinoma cancer cell line, was kindly provided by Prof. P. C. Yang (National Taiwan University) [33]. CL1-0 cells were cultured in RPMI1640 medium supplemented with 10% fetal bovine serum (FBS) and 1% antibiotics. Cell lines were cultured in 5% CO_2_ at 37 °C. The antibiotic concentration was 100 U/mL penicillin and streptomycin.

### 4.6. Fluorescence Lifetime Imaging Microscopy (FLIM)

The setup of the FLIM system consisted of a picosecond diode laser (laser power, 5 mW) with an emission wavelength of 470 nm (LDH470; PicoQuant, Berlin, Germany) and a ~70 ps pulse width for the excitation of *o*-BMVC under a scanning microscope (IX-71 and FV-300; Olympus, Tokyo, Japan). The fluorescent signal from the *o*-BMVC was collected using a 60 × NA = 1.42 oil-immersion objective (PlanApoN; Olympus, Tokyo, Japan) passing through a 550/88 nm bandpass filter (Semrock, Rochester, New York, NY, USA), followed by detection using a SPAD (PD-100-CTC; Micro Photon Devices, Bolzano, Italy), the time resolution of which was less than 50 ps FWHM. The fluorescence lifetime was recorded and analyzed using a time-correlated single-photon counting (TCSPC) module and software by mono-exponential curve fitting (PicoHarp 300; electrical time resolution was less than 25 ps; and SymPhoTime v5.3.2; PicoQuant, Berlin, Germany). FLIM images were constructed from pixel-by-pixel lifetime information. Time-gated FLIM images had set time windows at 2.4 ns to separate the image into two colors: white (decay time ≥ 2.4 ns) and red (decay time < 2.4 ns).

### 4.7. Confocal Microscopy

CL1-0 cells were treated with 1 μM Cy5-HT23 for different hours and were co-stained with 50 nM LysoTracker green DND-26 (Invitrogen, Waltham, MA, USA) for 15 min. Samples were washed twice with PBS and visualized using a confocal microscope (Leica TCS SP8; Leica, Wetzlar, Germany).

## 5. Conclusions

In this work, we first demonstrate that the intermolecular parallel G4s can be accumulated in the lysosomes of CL1-0 live cells. In addition, the important finding is the detection of the parallel G4s of HT23 in the lysosomes, indicating that the structural conversion of GROs from the nonparallel G4 structure to the parallel G4 structure is possible in CL1-0 live cells. Such a finding supports the prevailing view that the parallel G4 structure is likely the major form in live cells. In addition, the large differences in the G4 signals detected from the incubation of ss-CMA, ss-GTERT-d(FN), and ss-HT23 can provide new insights for the study of G4-lysosome interactions in the cells. It appears that the lysosome is a good organelle for the study of G4, and the exogenous GROs can be used as markers to reveal the composition and function of lysosomes.

## Data Availability

Not applicable.

## Supplementary Materials

The following supporting information can be downloaded at: https://www.mdpi.com/article/10.3390/ijms232415799/s1.
