# Supplementary material for: Structural Change from Nonparallel to Parallel G-Quadruplex Structures in Live Cancer Cells Detected in the Lysosomes Using Fluorescence Lifetime Imaging Microscopy
Source: Int J Mol Sci. 2022 Dec 13;23(24):15799. doi: 10.3390/ijms232415799 (PMC9779097; doi:10.3390/ijms232415799)
Supplement: Supplementary file 1 [file ijms-23-15799-s001.zip › ijms-2056532-supplementary.pdf]

Supporting Information

Structural Change from Nonparallel to Parallel G-Quadruplex Structures in Live Cancer Cells Detected in the Lysosomes Using Fluorescence Lifetime Imaging Microscopy

Ting-Yuan Tseng, Chiung-Lin Wang and Ta-Chau Chang *

Institute of Atomic and Molecular Sciences, Academia Sinica, Taipei 10617, Taiwan

* Corresponding author: Ta-Chau Chang (email: tcchang@pub.iams.sinica.edu.tw)

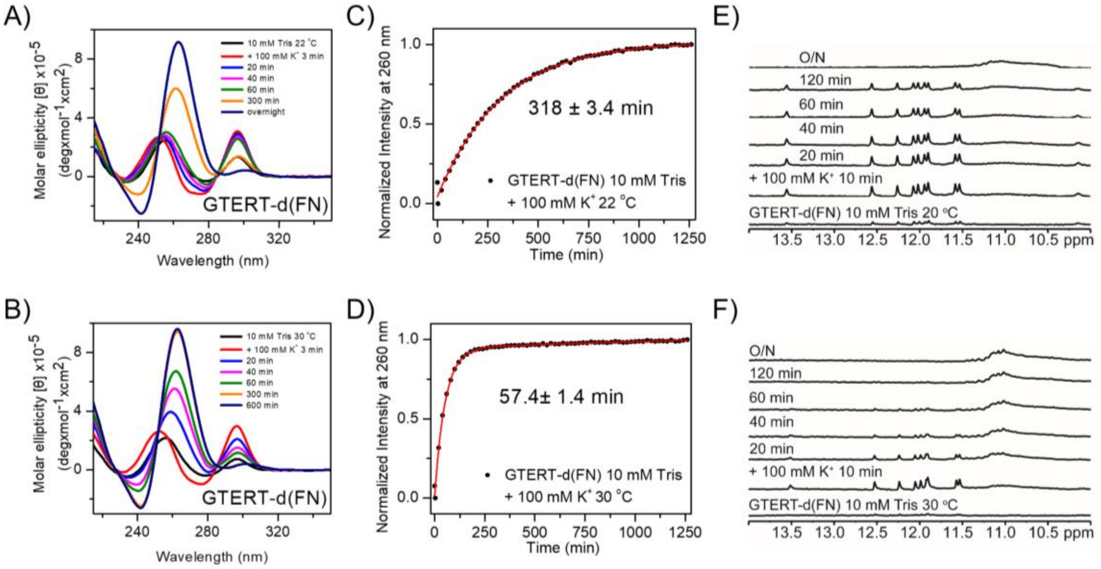

Figure S1. Temperature-dependent study of the structural conversion of GTERT-d(FN) in a K^+ solution. Time-dependent CD spectra of GTERT-d(FN) in a 100 mM K^+ solution at 22 ^{o}C (A) and 30 ^{o}C (B). The arising time of G4-2 signals obtained from a single exponential fitting at 265 nm is 318±3.4 min at 22 ^{o}C (C) and 57.4±1.4 min at 30 ^{o}C (D). Time-resolved imino proton spectra of GTERT-d(FN) recorded at 0, 10, 20, 40, 60, 120 min, and overnight after the addition of 100 mM K^+ at 22 ^{o}C (E) and at 30 ^{o}C (F).

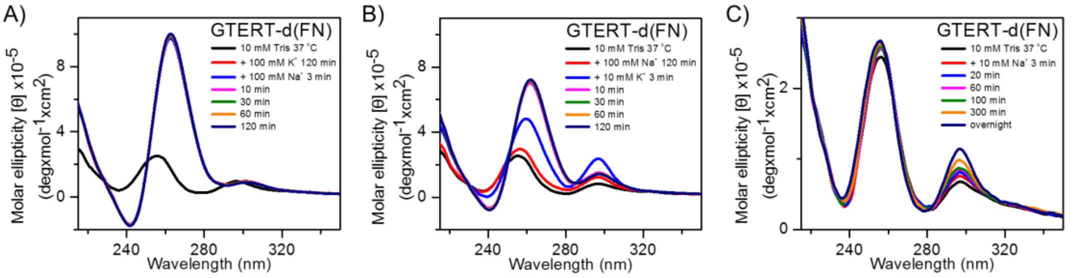

Figure S2. CD spectra of GTERT-d(FN) in a 100 mM K^+ solution for 2 h followed by the addition of 100 mM Na^+ for 3, 10, 30, 60, and 120 min (A). CD spectra of GTERT-d(FN) in a 100 mM Na^+ solution for 2 h followed by the addition of 10 mM K^+ for 3, 10, 30, 60, and 120 min (B). Time-dependent CD spectra of GTERT-d(FN) in a 10 mM Na^+ solution (C).

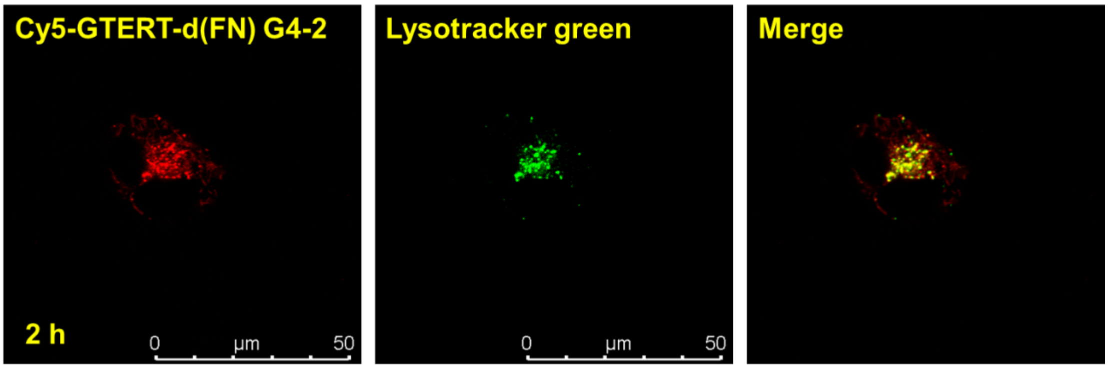

Figure S3. Confocal images of Cy5-GTERT-d(FN) G4-2 incubated with CL1-0 live cells for 2 h (left), and then stained by LysoTracker green at a concentration of 50 nM incubated with cells for 15 min (middle) together with their merges (right).
